# Handling Complaints Related to Diagnostic Faults Before Disciplinary Bodies: Analysis of French Ordinal Jurisprudence

**DOI:** 10.7759/cureus.79564

**Published:** 2025-02-24

**Authors:** Awatif Aheri, Mohammed Amine Daerqaoui, Fatima Belamine, Ahmed Belhouss, Hicham Benyaich

**Affiliations:** 1 Forensic Medicine, Ibn Rochd University Hospital, Casablanca, MAR

**Keywords:** diagnostic fault, disciplinary responsibility, ethical challenges in general practice, medical ethics, medicolegal disciplinary actions, ordinal jurisprudence

## Abstract

Introduction

Diagnostic faults are a major source of medical litigation, potentially engaging the disciplinary responsibility of physicians. The distinction between diagnostic fault and diagnostic error is often misunderstood, which can lead to unjustified sanctions or ignorance of ethical obligations. This study aims to clarify the concept of diagnostic fault as an ethical breach and to identify situations that may engage the physician’s disciplinary responsibility. It further highlights the ethical and medicolegal challenges posed by diagnostic errors, providing insights into their implications for deontological and legal frameworks.

Materials and methods

This is a descriptive and analytical retrospective study of 73 disciplinary procedures for diagnostic faults judged by the National Disciplinary Chamber of the French Medical Council between 2012 and 2020. Data were collected from the jurisprudence database of the National Council of the French Medical Council. Inclusion criteria included cases where a diagnostic fault was invoked. Cases in which no diagnostic fault or delay was alleged at the outset against the physician were excluded from the analysis. A descriptive statistical analysis was performed.

Results

Among the 73 cases reviewed by the disciplinary chamber, several involved multiple complainants. General practitioners were the most frequently implicated, accounting for 45% of cases. Of the cases examined, 33 (45%) were dismissed due to insufficient evidence or absence of fault. Delayed diagnoses (22%) and erroneous diagnoses (21%) emerged as the most frequently retained reasons. Finally, firm suspensions, ranging from 8 days to 2 years, were the most common sanction, imposed in 15 cases (21%).

Discussion

Diagnostic faults represent a significant portion of disciplinary litigation. Situations leading to these faults can be classified into three categories: lack of attention during diagnosis, absence of appropriate complementary investigations, and failure to consult a competent third party. Comparison with Moroccan legislation reveals differences in disciplinary procedures, particularly in terms of transparency and procedural safeguards.

Conclusion

A diagnostic fault is an ethical breach that can engage the physician’s disciplinary responsibility. A better understanding of ethical obligations and continuous training is essential to reduce these faults. Disciplinary procedures should be revised to include guarantees of a more transparent and equitable process.

## Introduction

In Morocco, the number of lawsuits filed against physicians has markedly increased over the past decade (2010-2020), reflecting heightened patient awareness and an evolving landscape of medical accountability. This trend is supported by both our forensic medicine department’s experience at Centre Hospitalier Universitaire (CHU) Ibn Rochd in Casablanca and the limited number of Moroccan studies on the subject, notably the study conducted by Boubess et al. in Rabat, published in 2022 [1]. Medical responsibility encompasses various forms, including civil, criminal, administrative, and disciplinary liability. While civil and criminal aspects have been extensively studied, disciplinary responsibility, particularly regarding diagnostic fault, remains largely underexamined [2]. Moreover, no comprehensive statistics are available on disciplinary sanctions against physicians. Due to the unavailability of published Moroccan ordinal jurisprudence, this study relies on French ordinal jurisprudence to address these gaps and provide a broader understanding of the subject.

Diagnostic fault, despite being under intense judicial scrutiny, is not consistently recognized as an ethical violation that may lead to disciplinary accountability [2]. It differs from a mere diagnostic error by involving a verified breach of the duty of care - such as negligence, imprudence, or demonstrable incompetence - rather than an unavoidable medical uncertainty [3]. As such, it can be regarded as an ethical violation, contravening the principles of beneficence and non-maleficence and potentially undermining patient autonomy [4]. Moreover, although various studies have examined legal accountability in healthcare, comprehensive data on disciplinary sanctions remains scarce, limiting our overall understanding of these faults and revealing a gap in the literature [5-6].

In this context, this study pursues several specific objectives. First, it aims to clarify the concept of diagnostic fault by rigorously distinguishing it from diagnostic error to provide a more precise framework for assessing medical responsibility. Second, it seeks to identify recurring scenarios leading to disciplinary sanctions by analyzing the reasons invoked before disciplinary bodies. Third, it compares disciplinary procedures between France and Morocco to examine structural differences and identify potential improvements that could be applied to the Moroccan system. By exploring these three dimensions, this study aims to contribute to a better understanding of disciplinary responsibility related to diagnostic faults and to shed light on potential regulatory reforms in medical governance.

## Materials and methods

This study employed a descriptive and exploratory retrospective design, focusing on disciplinary cases involving general practitioners and specialists in France. The analysis targeted proceedings brought before the disciplinary bodies of the French Medical Council for alleged diagnostic faults, regardless of the case outcomes. All data were extracted from the publicly accessible jurisprudence database maintained by the French National Council of the Order of Physicians [7], and the study examined cases adjudicated between 2012 and 2020. This period was selected to cover a significant timeframe (nearly a decade) and to ensure the representativeness of recent disciplinary cases in France.

Cases were included if they involved proceedings before the National Disciplinary Chamber of the French Medical Council and explicitly cited a diagnostic fault as the reason for the complaint. Cases unrelated to diagnostic faults, those with incomplete or inaccessible files, or cases in which no diagnostic fault or delay was alleged at the outset against the physician were excluded from the analysis. These cases did not meet the study’s objective, which focused on instances where disciplinary action was taken due to an alleged or confirmed diagnostic fault. Data collection involved a keyword search within the jurisprudence database using terms such as “diagnostic,” “fault,” “error,” and “negligence.” Of the 80 initially identified decisions, 73 met the inclusion criteria. Key variables extracted included the complainant’s status (e.g., patient, beneficiary, Departmental Council, National Council, or Primary Health Insurance Fund), the physician’s specialty (general practitioner or specialist), the grounds for the complaint (e.g., delayed diagnosis, erroneous diagnosis, or lack of complementary examinations), and the disciplinary decisions (e.g., dismissal, warning, reprimand, or suspension). To ensure data accuracy, a standardized exploitation sheet was used, including key variables such as case number, complainant, physician specialty, allegations, and disciplinary decisions. Additionally, cases were independently reviewed by two researchers to enhance reliability and minimize potential errors.

The collected data were systematically entered and analyzed using Microsoft Excel (Microsoft Corporation, Redmond, WA, US). Since this study is primarily descriptive, no inferential statistical tests were applied. Frequencies and percentages were calculated to summarize the variables under study and provide a comprehensive overview of the disciplinary cases. A clinical trial number was not applicable. This study did not involve human subjects or interventional research requiring prior ethics committee approval. Ethical considerations were carefully addressed, as this study relied exclusively on publicly available and anonymized data from the jurisprudence database of the French National Council of the Order of Physicians. Since the data were openly accessible and devoid of personal or confidential information, no prior approval from an ethics committee was required. Since this study relies exclusively on publicly available and anonymized data from the jurisprudence database of the French National Council of the Order of Physicians, no prior approval from an ethics committee was required. However, ethical considerations were carefully addressed, and the study complies with international ethical standards, particularly the Council for International Organizations of Medical Sciences (CIOMS) guidelines [8] and the Helsinki Declaration [9], which govern the ethical use of public data.

## Results

Complainants’ status

A total of 86 complaints were filed across 73 disciplinary cases, as some cases involved multiple complainants. The majority of complaints, 63 in total (73%), were initiated by patients or their beneficiaries, highlighting their central role in initiating proceedings. Departmental Councils (DC) accounted for 15 complaints (17%), 9 of which were filed jointly with patients. Additionally, the National Council of the Medical Order (NCMO) submitted five complaints (6%), with two of these associated with patient complaints. Finally, the Primary Health Insurance Fund (PHIF) introduced three complaints (4%), including one submitted in conjunction with a patient.

Specialty of prosecuted physicians

General practitioners were the most frequently implicated, accounting for 33 cases (45%). Among specialists, gynecologists were involved in 7 cases (10%), radiologists in 5 cases (7%), general surgeons in 4 cases (5%), ophthalmologists in 4 cases (5%), and anesthesiologists in 3 cases (4%). Various other specialties, including internal medicine, psychiatry, and cardiology, were implicated in 17 cases (24%). These findings emphasize the predominance of general practitioners in disciplinary complaints related to diagnostic faults. This trend may be explained by the broad scope of general practice, which requires managing a wide spectrum of medical conditions without the benefit of specialization. Additionally, general practitioners serve as the primary point of contact in the healthcare system, often working in resource-limited settings with restricted access to specialized consultations and diagnostic tools. These factors increase the likelihood of diagnostic challenges and, consequently, the risk of disciplinary complaints.

Disciplinary decisions

In 45% of the cases (33/73), the complaints were dismissed due to insufficient evidence or absence of fault. Among the remaining 40 cases (55%) that resulted in sanctions, 22 cases (30%) confirmed the initial decision, 10 cases (14%) led to a mitigation of the initial sanctions, and 2 cases (3%) resulted in aggravated sanctions upon reassessment. Additionally, 6 cases (8%) involved other ethical violations unrelated to diagnostic faults such as billing abuse or neglect in maintaining medical records.

These results highlight that nearly half of the disciplinary cases were dismissed while the other half led to sanctions, demonstrating the diversity of outcomes in the disciplinary process related to diagnostic faults.

Detailed disciplinary sanctions and their durations

Of the 73 cases reviewed by the disciplinary chamber, 33 (45%) were dismissed without further action due to lack of evidence or absence of fault. Among the 40 cases that resulted in sanctions, a variety of disciplinary actions were imposed. Firm suspensions, ranging from 8 days to 2 years, were the most common, affecting 15 cases (21%). Warnings were issued in 9 cases (12%) while suspended suspensions with specific conditions were applied in 5 cases (7%). Four cases (5%) involved firm suspensions combined with mandatory training requirements. Reprimands were recorded in three cases (4%) while removal from the medical register occurred in two cases (3%). Additionally, two cases (3%) involved prohibitions on providing care to insured persons, with durations of four and eight months, respectively.

These findings emphasize the diversity of sanctions applied, with firm suspensions being the most frequently imposed measure among sanctioned cases. This reflects the gravity of certain offenses and the commitment to upholding professional standards.

Reasons for complaints 

The most frequently cited reasons for complaints were delayed diagnosis and erroneous diagnosis, each accounting for 34 complaints (29%). Other reasons included missing complementary exams (21 complaints, 18%), insufficient clinical examination (19 complaints, 17%), failure to consult a competent third party (7 complaints, 6%), and insufficient questioning (1 complaint, 1%).

It is important to note that the total number of grievances (116) exceeds the number of unique complaints (73), as a single complaint could involve multiple reasons. This cumulative approach highlights the complexity and multifaceted nature of diagnostic faults identified in disciplinary cases.

Reasons retained by the Disciplinary Chamber 

Of the 73 cases reviewed by the disciplinary chamber, 33 (45%) were dismissed due to insufficient evidence or absence of fault. Among the 40 sanctioned cases, a total of 57 faults were identified, as some cases involved multiple infractions.

Delayed diagnoses were the most frequent category, accounting for 16 cases (22% of all cases reviewed by the disciplinary chamber). Erroneous diagnoses followed with 15 cases (21%). Insufficient clinical examination and missing complementary exams were retained in 10 cases each (14%). Failure to consult a competent third party was identified in five cases (7%), while insufficient questioning was noted in one case (1%).

These findings highlight the diversity of faults retained by the disciplinary chamber and their significance in final decisions.

Other ethical breaches

In six cases, no diagnostic fault was retained; however, sanctions were imposed for other ethical violations, including inappropriate treatment, negligence, failure to provide medical records, billing abuse, and inadequate information. In 10 cases, additional ethical breaches (professional insufficiency, inappropriate treatment, negligence) were sanctioned alongside the diagnostic fault.

## Discussion

Given the lack of publicly available Moroccan disciplinary jurisprudence, this study primarily relied on French jurisprudence to analyze disciplinary accountability related to diagnostic faults. The choice of France is justified by the structured and transparent nature of its disciplinary system, which provides detailed published rulings, serving as a valuable reference for understanding medico-legal principles. Although Moroccan data were not directly accessible, a comparative approach was adopted to highlight key differences in disciplinary frameworks, particularly regarding procedural safeguards and transparency. This analysis aims to provide insights into potential improvements for the Moroccan system while contextualizing the findings within a broader legal and ethical perspective.

Discussion of the results

Our study shows that diagnostic faults account for approximately 55% of the disciplinary cases reviewed by the French National Disciplinary Chamber. This figure highlights a particular focus on the quality of care, especially the rigor of clinical reasoning, within an ordinal framework where published rulings serve a pedagogical function through jurisprudence.

Profile of Complainants

Patients and their beneficiaries filed the majority of complaints (73%) in our study, significantly exceeding the 56% reported in 2019 for all complaints [10]. This overrepresentation underscores the heightened sensitivity of patients to diagnostic faults, which are often perceived as severe lapses. These results may reflect growing patient expectations in the context of rapidly evolving medical knowledge.

Comparison With French Statistical Data

Infractions related to care quality (including diagnosis) accounted for nearly 50% of all cases in the 2019 activity report [10]. In comparison, 45% of diagnostic-fault complaints in our study were dismissed versus an overall dismissal rate of 33.5% in 2019 for all complaints. This discrepancy may stem from stricter disciplinary assessments of diagnostic faults due to their potential severity and impact or contextual factors such as the nature of the cases and their specific circumstances.

Outcome of Complaints and Sanctions

The sanctions imposed in our study align broadly with the 2019 findings, with some differences. Firm suspensions were slightly more frequent in our study (21% compared to 16.5% in 2019) while warnings were less common (12% versus 17.5%). Removals from the medical register were consistent across both datasets at 4% [10]. These variations may reflect differences in the types of cases reviewed or adjudication practices. However, the overall distribution of sanctions remains stable, highlighting the robustness of the disciplinary framework while accommodating case-specific considerations.

General discussion

 *Comparison of Disciplinary Procedures in France and Morocco*

The absence of publicly accessible activity reports in Morocco poses challenges for direct international comparisons. Nevertheless, several key differences between the disciplinary procedures in France and Morocco emerge, showcasing their distinct approaches to managing professional accountability and resolving conflicts.

Initiators of complaints: In France, a wide range of entities can initiate complaints, including health insurers, patient advocacy groups, ministers, prefects, prosecutors, unions, practitioner associations, and national or departmental councils of the medical order. In Morocco, only patients, their representatives, heirs, the administration, unions, professional associations, and council presidents can file complaints under Article 73 of law 08-12. Health insurers and patient advocacy groups are excluded in Morocco.

Attempted prior conciliation in France vs. no conciliation process in Morocco: In France, the medical disciplinary procedure generally includes a preliminary conciliation stage aimed at resolving disputes before they are brought before a judicial body. This approach provides an opportunity for dialogue and mediation between the parties, potentially avoiding lengthy and contentious proceedings [11]. In contrast, Morocco does not formally incorporate such a preliminary conciliation mechanism in its disciplinary procedures. Instead, complaints are directly reviewed by the relevant authorities without any attempt at amicable resolution [12]. This difference highlights the fundamental variations between the two systems, reflecting distinct approaches to managing disciplinary conflicts.

Complaint admissibility: In France, the chair of the disciplinary chamber can dismiss unfounded complaints before investigation. This significant authority of the chairs of disciplinary chambers to manage complaints is a common practice in several European legal systems [13]. In Morocco, a commission evaluates the admissibility, identifies the presumed faults, gathers evidence, and prepares the file for the disciplinary hearing.

Sanctions and specialized sections: In France, disciplinary measures include a wider range of options such as prohibitions on treating insured persons and mandatory training in cases of professional insufficiency. Morocco has no equivalent, and sanctions are generally of shorter duration. For example, firm suspensions can last up to three years in France, while in Morocco, they are limited to a maximum of one year. This disparity reflects differing approaches to disciplinary severity and professional accountability between the two systems. This disparity reflects differing approaches to disciplinary severity and professional accountability between the two systems, as highlighted in discussions of disciplinary actions in Morocco [14].

Appeal procedures: The French and Moroccan systems differ significantly in the handling of disciplinary appeals. In France, decisions can be challenged before the Conseil d’État, with additional mechanisms, such as revision or rectification of material errors, which allow judgments to be amended when new evidence emerges. Furthermore, opposition is permitted for default rulings [7].

In Morocco, the options are more limited. Appeals are exclusively handled by the administrative chamber of the Court of Cassation, without extraordinary remedies like those available in France. Opposition is restricted to decisions made by regional disciplinary formations [15].

These differences reflect a more comprehensive approach in France, offering greater safeguards to correct judicial errors through the mechanisms of the Conseil d’État, as highlighted in French jurisprudence [16].

Despite these procedural differences, both France and Morocco share common principles in their approach to disciplinary accountability. In both systems, the objective remains the protection of patients and the regulation of medical practice through professional oversight. Additionally, the role of medical councils as key disciplinary bodies is a shared feature, ensuring that decisions are made within a framework that prioritizes professional ethics and patient safety [3,17].

Implications of Stringent Disciplinary Standards

While strict disciplinary measures reinforce medical accountability and patient safety, they also carry implications for clinical practice. Excessively stringent disciplinary standards may contribute to defensive medicine, where physicians order additional, sometimes unnecessary, tests to mitigate litigation risks [18,19]. This could lead to increased healthcare costs and longer diagnostic delays. Furthermore, harsh sanctions may deter professionals from reporting diagnostic challenges or seeking second opinions, potentially hindering a culture of learning and improvement in medical practice [20]. A balanced approach, incorporating both accountability and support mechanisms, is essential to maintaining patient trust while safeguarding the integrity of medical decision-making.

Diagnostic Fault: Distinction and Responsibility

Distinguishing error from fault: A simple diagnostic error, arising from the complexity of pathologies or challenging circumstances (e.g., emergency care, comatose patient), is not automatically considered a fault [21,22]. A fault requires evidence of negligence, imprudence, or inadequate use of diagnostic resources (patient interview, clinical exam, complementary tests, and consultation with specialists) [3]. The recognition that diagnostic complexity can lead to errors without constituting a fault is not new. Early French jurisprudence had already acknowledged that not all diagnostic errors are blameworthy, as illustrated by historical rulings that considered the inherent difficulty of accurate diagnosis under certain conditions French jurisprudence has highlighted that physicians cannot expect patients to spontaneously provide all necessary information, as modesty, low intellectual levels, or misconceptions about the relevance of certain details may interfere [23]. Furthermore, diagnostic faults can stem from the absence of a clinical examination or from a hasty and superficial assessment. Errors become blameworthy when they result from carelessness or negligence during the examination, which involves observing the patient’s general condition, measuring vital signs, and conducting a systematic physical examination [24].

Civil, criminal, and disciplinary liability: A diagnostic fault can trigger civil liability (compensation), criminal liability (homicide or unintentional harm) [25], or disciplinary liability (warning, suspension, or revocation). The severity of the sanction is determined by both the seriousness of the harm and the degree of negligence or deviation from professional standards [26].

Ethical and Systemic Factors Influencing Diagnostic Faults

The ethical implications of diagnostic faults extend beyond individual clinical decisions, encompassing broader concerns related to negligence, malpractice, and patient trust. A diagnostic fault differs from an unavoidable error, as it involves a breach of the duty of care, often characterized by negligence, imprudence, or inadequate use of available diagnostic tools. This distinction is essential, as it directly impacts legal accountability and professional integrity.

Several factors contribute to diagnostic faults, which can be classified into three main categories: systemic factors, patient-related factors, and physician-related factors. Systemic factors include high workload, resource constraints, and inefficiencies within healthcare systems, which can lead to time pressure and suboptimal diagnostic decisions [27]. Patient-related factors, such as limited adherence to medical recommendations, communication barriers (language proficiency, low health literacy), and complex medical histories, can also complicate the diagnostic process [28]. Lastly, physician-related factors, such as fatigue, burnout, and cognitive overload, play a crucial role in diagnostic performance and can increase the risk of errors [29].

Diagnostic faults are not solely influenced by medical or technical factors but are also shaped by cultural and societal elements that determine how medical errors are perceived and managed. The doctor-patient relationship varies across contexts: in some cultures, physicians are seen as unquestionable authorities, limiting patient challenges to medical decisions and reducing complaint rates. In contrast, in societies that prioritize transparency and patient rights, there is a stronger demand for explanations, often leading to increased medical litigation [30]. Moreover, access to disciplinary and judicial mechanisms plays a crucial role. While some countries have institutional frameworks that facilitate complaint procedures, others impose administrative, financial, or social barriers that discourage patients from pursuing legal action. The culture surrounding medical error reporting also influences how diagnostic faults are addressed. In certain systems, proactive disclosure is encouraged to improve patient safety, whereas, in others, fear of sanctions may deter such reporting [31]. These variations also affect medical practice trends, particularly in highly litigious environments, where defensive medicine - characterized by excessive reliance on diagnostic tests to mitigate legal risks - becomes more prevalent. As a result, these cultural and societal factors shape the perception of diagnostic faults, patient responsiveness, and physician adaptation, highlighting the need for a balanced framework that ensures both patient protection and professional security.

When comparing these observations with international studies, similar trends are observed in countries with robust medico-legal systems, such as the United States and the United Kingdom, where the increasing emphasis on patient autonomy and legal accountability has led to heightened scrutiny of diagnostic decisions [32,33]. However, in countries with developing healthcare infrastructures, factors such as resource constraints and less stringent regulatory frameworks often limit the ability to systematically address diagnostic faults [34,35]. These differences highlight the need for a balanced approach that ensures patient safety while mitigating the risks of excessive judicialization, which may inadvertently encourage defensive medicine [36].

Recommendations and prevention

Continuing Education

Gaps in continuing education or outdated knowledge significantly increase the risk of diagnostic errors. Establishing a national mechanism to organize and monitor continuing education is essential to ensure that medical practices evolve alongside scientific advancements.

Material Resources

Poor working conditions, lack of equipment, and heavy workloads can contribute to errors. Healthcare authorities should provide medical professionals with sufficient access to modern infrastructure and adequate financial resources to ensure optimal performance.

Collaboration and Multidisciplinary Work

Physicians must acknowledge their limitations and actively seek consultations with colleagues or engage in multidisciplinary board discussions. Tools such as telemedicine and multidisciplinary team meetings (MDTs) should be more widely adopted in practice.

Improving Transparency and Accessibility of Disciplinary Data

A more transparent disciplinary system would reinforce both public trust and medical accountability. The digitization of disciplinary records, while ensuring the anonymity of complainants and physicians, could serve as a pedagogical tool for healthcare professionals and inform patients about medical standards. Additionally, adopting a French-inspired model of publishing annual disciplinary reports would enhance institutional transparency, allowing professionals and the public to understand how medical responsibility is enforced. This approach would help create a data-driven system where past cases contribute to professional education and policy improvement.

Guidelines for Best Practices

Develop concise, accessible recommendations prioritizing non-invasive techniques while enhancing the role of general practitioners within care pathways.

Strengthening Multidisciplinary Collaboration and Diagnostic Assistance Technologies

Enhancing multidisciplinary collaboration is crucial to reducing diagnostic faults. Encouraging systematic interdisciplinary consultations, including case reviews in MDTs, can improve diagnostic accuracy and prevent faults related to isolated clinical decisions. Integrating artificial intelligence (AI)-assisted diagnostic tools and clinical decision support systems (CDSS) can further help reduce uncertainty and standardize medical reasoning.

Moreover, expanding telemedicine programs can facilitate timely expert opinions in complex cases, particularly in remote areas where access to specialists is limited. National healthcare policies should support these initiatives by investing in secure telemedicine platforms, developing standardized protocols for remote diagnostics, and ensuring adequate training for healthcare professionals.

Limitations of the study

This study presents several methodological limitations that should be considered when interpreting the results.

Selection Bias and Underreporting

The study is based exclusively on publicly available jurisprudential data from the database of the French National Medical Council. Not all disciplinary decisions are necessarily published, which may introduce underreporting or selection bias. Certain unpublished cases may contain nuances that could refine our findings.

Sample Size and Representativeness

Although the study covers an eight-year period (2012-2020), the sample of 73 cases remains relatively limited. While it provides general trends on disciplinary accountability related to diagnostic faults, it does not allow for broad generalization to all disciplinary decisions.

Lack of Direct Comparison With Morocco

The inability to access Moroccan disciplinary rulings limited our ability to conduct a direct comparative analysis between the two countries. Consequently, the structural differences identified are based primarily on an analysis of regulatory texts and existing literature, which constitutes a limitation for interpreting the results in a Moroccan context.

Unmeasured Confounding Factors

Certain institutional and contextual factors, such as differences in how faults are reported, defense strategies of accused physicians, or specific disciplinary practices in different jurisdictions, could not be quantified. These elements may influence disciplinary decisions and represent unmeasured confounding variables in our analysis.

Descriptive Analysis Without Advanced Statistical Testing

Due to the relatively small sample size, the study relied solely on descriptive analysis of frequencies and percentages. No comparative statistical tests (e.g., chi-square test and multivariate analysis) were performed, which limits the robustness of the inferences drawn.

Despite these limitations, this study represents a comprehensive first analysis of diagnostic faults in disciplinary proceedings, shedding light on critical ethical and regulatory aspects that require further scrutiny. Our findings provide a solid foundation for future research, potentially integrating a more diverse dataset and advanced statistical analyses to deepen the understanding of diagnostic faults in various medical and legal contexts.

## Conclusions

Diagnostic faults represent a significant share of disciplinary proceedings, underlining the importance of a rigorous and continually updated clinical approach. The high proportion of unfavorable outcomes for physicians highlights the vigilance of disciplinary bodies in upholding care standards.

Comparison with Moroccan procedures reveals the need for a more transparent, cohesive disciplinary system, along with systematically published decisions that offer educational feedback. Reinforcing continuous training, improving material resources, and promoting interdisciplinary collaboration are crucial for reducing diagnostic errors. Ultimately, these measures may strengthen mutual trust between physicians and patients, thereby increasing the overall effectiveness of healthcare services.
